# Open Repair for Patent Ductus Arteriosus Aneurysm in an Adult

**DOI:** 10.3400/avd.cr.21-00087

**Published:** 2021-12-25

**Authors:** Yuya Kise, Yukio Kuniyoshi, Syotaro Higa, Mizuki Ando, Tatuya Maeda, Hitoshi Inafuku, Moriyasu Nakaema

**Affiliations:** 1 Department of Thoracic and Cardiovascular Surgery, Graduate School of Medicine, University of the Ryukyus, Nishihara, Okinawa, Japan; 2 Department of Cardiovascular Surgery, Urasoe General Hospital, Urasoe, Okinawa, Japan

**Keywords:** ductus arteriosus aneurysm, adult, open repair

## Abstract

Ductus arteriosus aneurysm (DAA) is rarely encountered in adults. There have been several hypotheses regarding its origin and potential indications for intervention in asymptomatic cases. If left untreated, rupture, compression of surrounding organs, and serious complications due to thromboembolism may occur, and aggressive surgical intervention appears desirable for patients who can tolerate surgery. We report a case involving a 30-mm, saccular, patent DAA that was incidentally discovered in a 49-year-old man on computed tomography. Open repair was performed by femorofemoral bypass assistance, which allowed decompression of the aorta and aneurysm and successful closure of the aortic and pulmonary artery ends.

## Introduction

Ductus arteriosus aneurysm (DAA) is a rare disease, and the underlying mechanisms have not yet been fully elucidated.^1)^ In addition, few reports have described patency of the pulmonary arterial side in adult DAA cases.^1)^ DAA is typically found either incidentally on examination of imaging^2)^ or when the patient develops hoarseness. However, serious complications such as compression of the bronchus and esophagus, embolism due to thrombosis in the aneurysm, and rupture can occur, leading to the diagnosis of DAA.^1,3)^ We report a case of patent DAA in an adult that was detected incidentally and treated by open repair with femorofemoral (FF) bypass.

## Case Report

**Figure 1:**
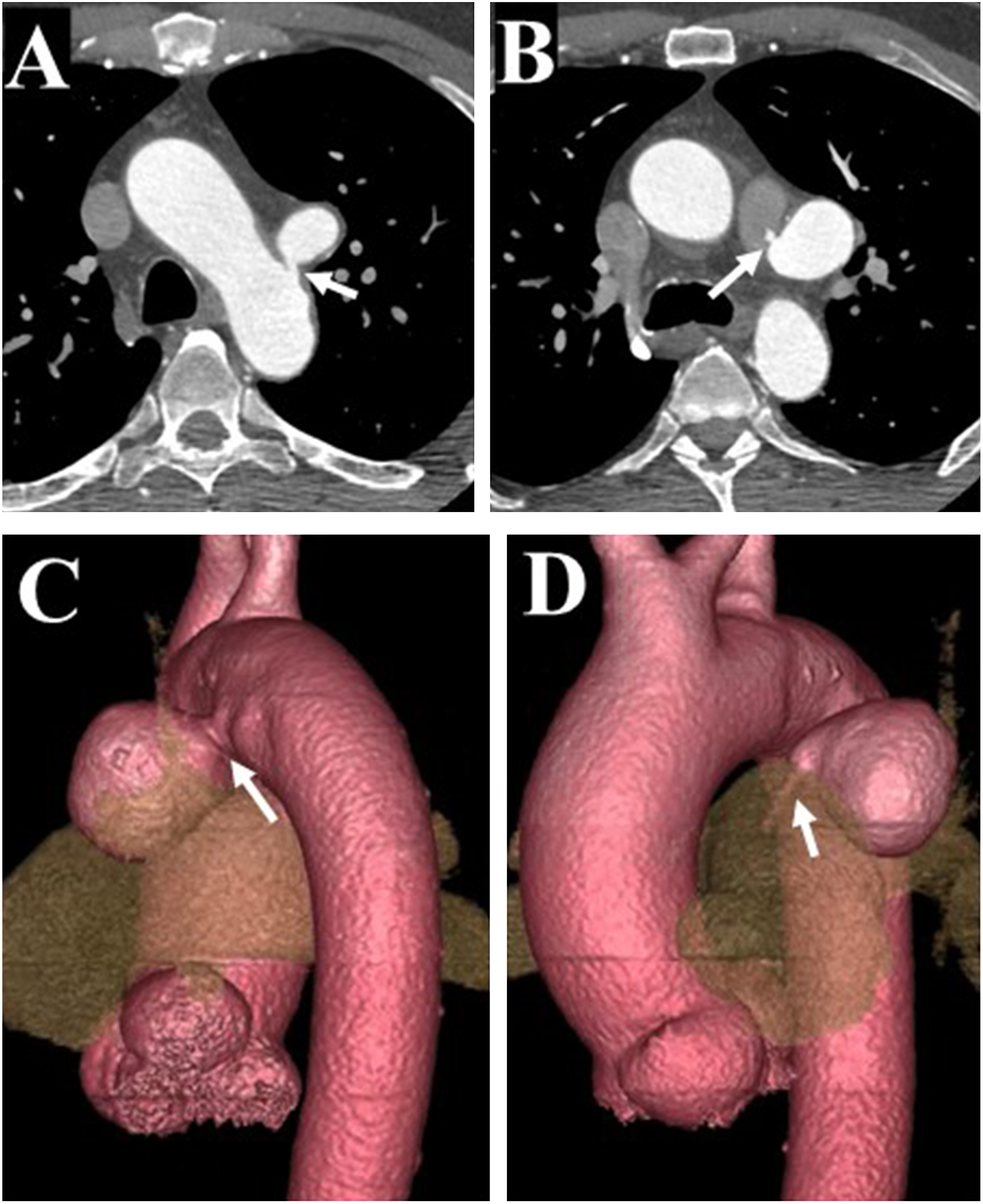
Fig. 1 Preoperative computed tomography. Fistula from the minor curve of the aortic arch to the aneurysm is confirmed (**A**, **C**; white arrow). Fistula from the aneurysm to the main pulmonary artery is confirmed (**B**, **D**; white arrow).

**Figure 2:**
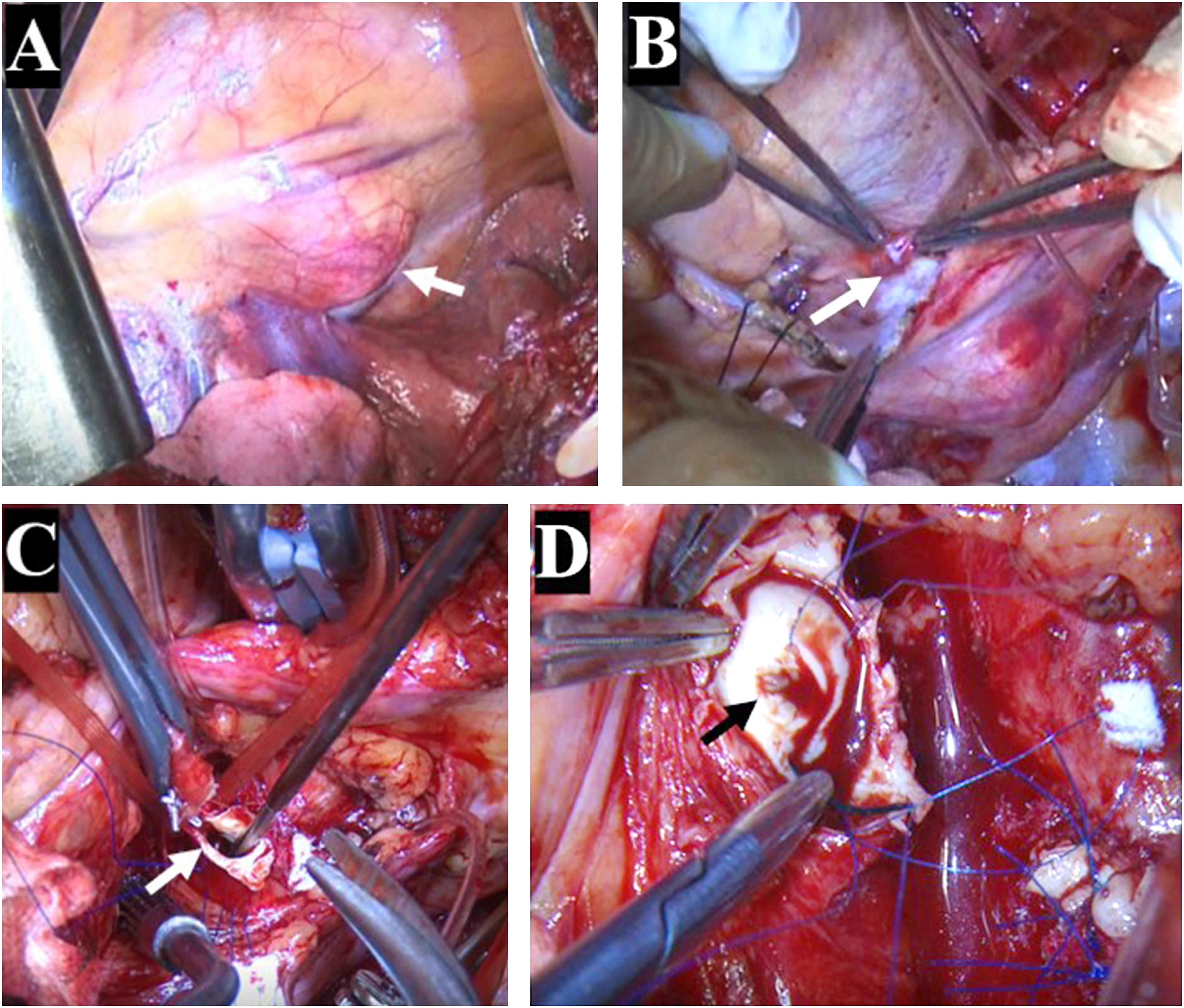
Fig. 2 Intraoperative photography. Ductus arteriosus aneurysm (DAA) is observed in the central visual field via left third intercostal posterolateral thoracotomy (**A**; white arrow). After pericardiotomy, the junction between the main pulmonary artery and DAA is confirmed (**B**, white arrow). The orifice on the aortic side is closed with a 4-0 Prolene mattress suture and felt strip (**C**, white arrow). After opening the aneurysm, the orifice on the pulmonary arterial side is closed with a 4-0 Prolene mattress suture and felt strip (**D**, black arrow).

A 49-year-old man with no medical history was found to have a continuous heart murmur in a general medical examination. He underwent echocardiography in the Department of Cardiology. Shunt flow from the aortic arch to the main pulmonary artery was observed, and patent ductus arteriosus (PDA) was identified. Contrast-enhanced computed tomography (CT) revealed a saccular aneurysm connected to the PDA, and the patient was referred to our department for treatment. No chest pain, hoarseness, or dysphagia were identified, and detailed CT examination revealed an arterial sac opening 6.4 mm in diameter from the ventral part of the distal aortic arch, resulting in a 30-mm saccular aneurysm. A 4.8-mm-diameter outflow was observed in the main pulmonary artery (PA) (**Fig. 1**). The artery of Adamkiewicz originated from the left 9th segmental artery, and pulmonary blood flow/systemic blood flow ratio was 1.2. We chose to perform open repair because we considered that the orifice needed to be closed not only on the aortic side but also on the pulmonary side. As relatively little atherosclerosis and calcification were present around the aneurysm, cross-clamping and simple closure were estimated to be safe to perform. A double-lumen tube was intubated under general anesthesia. Pressure lines were secured in both the right radial and left femoral arteries, then motor-evoked potential (MEP) monitoring was performed in preparation for descending thoracic aortic clamping. In the right lateral decubitus position, the aneurysm was located in the central visual field with the approach via left third intercostal posterolateral thoracotomy (**Fig. 2A**). The pericardium was opened in an upper pericardiotomy, and the junction between the main PA and aneurysm was confirmed (**Fig. 2B**). The descending thoracic aorta (Th4 level), left subclavian artery, and aortic arch (Zone II) on the proximal side of the aneurysm were taped. After administering 300 units/kg of heparin, partial extracorporeal circulation (ECC) was initiated by right femoral arterial infusion (FEMII20Fr; Edwards Lifesciences, Irvine, CA, USA), and a long 28-Fr cannula for venous drainage was placed from the right femoral vein to the right atrium. After reducing systolic blood pressure to 60 mmHg by increasing drainage on the side of the ECC to reduce both aortic pressure and intra-aneurysmal pressure, cross-clamping was performed at the aortic arch (Zone II), descending thoracic aorta (Th4 level), and left subclavian artery. The center of the aneurysm was then clamped using Satinsky forceps, and an incision was made between the aneurysm and orifice of the distal aortic arch. The orifice diameter was 7 mm, and the surrounding vascular wall demonstrated good properties (**Fig. 2C**); thus, a 4-0 polypropylene mattress suture with a felt strip was applied with three stitches to firmly close the orifice. When passing the needle, we paid attention to avoid damage to the recurrent nerve running just below the aneurysm. Next, when the aneurysm was opened toward the PA side, a 5-mm-diameter orifice was confirmed in the main PA (**Fig. 2D**). This orifice was also closed with two stitches of 4-0 polypropylene. The partial ECC time was 36 min, and the operation time was 3 h 40 min. No decrease in MEP amplitude was observed intraoperatively. The patient was discharged on postoperative day 14 with no complications.

## Discussion

DAA is often diagnosed by detailed echography either fetally (1.5%–2.2%) or neonatally (8.1%),^4)^ but the frequency in adults is unknown, and detailed reports are still lacking.^1,3,4)^ However, aneurysm of a ductal diverticulum with a closed PA end may be judged as a thoracic aortic aneurysm, and around 5% of thoracic aortic aneurysms in adults are reportedly derived from DAA.^5)^

There have been several hypotheses with regard to the pathogenesis of DAA. The following three theories are influential in terms of the congenital process: 1) Delayed closure of the ductus arteriosus results in the PA side closing first, then for some reason, the aortic side does not close and becomes a diverticulum.^1,4–6)^ 2) Cytolytic necrosis and mucoid degeneration of the media layer occur in the wall of the ductus arteriosus.^1,4)^ 3) Caused by connective tissue disorders, such as Marfan and Ehlers–Danlos syndromes.^1,4)^ Acquired formation has been reported as aneurysmal formation after PDA ligation in infancy. This is attributed to the cut made through the wall during ligation and the ligature being distant from the aorta and/or exhibiting expansion following stenosis.^1,7)^

DAA is often detected incidentally on imaging^2)^ or in patients presenting with hoarseness, but it is also sometimes encountered following severe complications, such as rupture, erosion (of the bronchi or esophagus), infection (arteritis or endocarditis), or thromboembolic events.^1,3)^ In neonates and infants, if no complications arise, approximately 98% of cases regress after 2 months of follow-up, but this tendency toward resolution is reportedly weaker for aneurysm diameters ≥7 mm.^8)^ For adults, while therapeutic intervention is considered appropriate for symptomatic cases, only a small number of asymptomatic cases have been encountered; thus, clear indicators are currently lacking. One opinion is that surgical intervention with due consideration of the attendant risk is desirable, because the risk of rupture increases with aneurysmal lesions ≥3 cm in diameter, even without symptoms.^9)^ On the other hand, considering that many adult DAA cases were found to be ruptured and that saccular aneurysms are constantly under aortic pressure, another opinion is that aggressive intervention should be performed in cases that can be treated as soon as they are found.^1,2,5)^

In open repair, both a posterolateral incision and an approach by median sternotomy are selected, then direct or patch closure of the fistula is performed via partial clamping or with the assistance of FF bypass. Some reports have described prosthetic graft replacement under deep hypothermic circulatory arrest.^5)^ A wide opening to the aneurysm may represent a good indication for surgical intervention, whereas calcifications make direct closure more difficult. Due to recent progress in endovascular repair devices, radical treatment with stent grafts and/or vascular plugs has also yielded good results in elderly patients.^10)^ In particular, thoracic endovascular aortic repair may be selected more aggressively for aneurysms of the ductus arteriosus diverticulum, in which the PA side is occluded. In the present case, we were concerned about leaving the PA opening, particularly in terms of thrombus growth expansion to the PA and the continuous pressure load on the aneurysm. With the assistance of FF bypass, the risk of pressure damage during aortic treatment can be avoided by maintaining circulation and reducing both aortic and aneurysmal pressure. Moreover, recurrent nerve injury due to the side clamp procedure could also be avoided. The PA fistula was also closed with a good field of view. In some adult cases, the aortic wall becomes hardened and calcified due to atherosclerotic changes. Therefore, safe procedures should be selected on a case-by-case basis using various available methods.

## Conclusion

We encountered an adult case of PDA aneurysm that was discovered incidentally. Given the availability of various surgical procedures, selection of the appropriate treatment method for each case is desirable.
